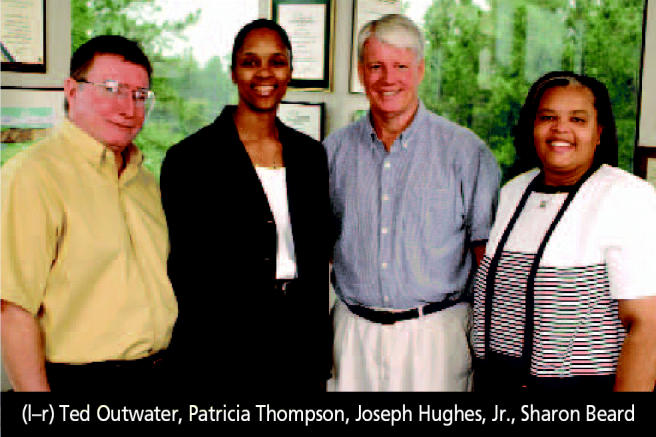# Worker Education and Training Branch

**Published:** 2004-09

The Worker Education and Training Branch (WETB) plans and administers grants, contracts, cooperative agreements, and interagency agreements to help organizations develop institutional competency to train hazardous waste workers and emergency responders. The WETB staff has worked to develop specific initiatives to support the programmatic functions of the branch.

The WETB is responsible for the Worker Education and Training Program (WETP), a training grants program established under the Superfund Amendments and Reauthorization Act of 1986. The mission of the WETP is to support the development of a network of nonprofit organizations that are committed to protecting workers and their communities by creating and delivering high-quality, peer-reviewed safety and health curricula to train hazardous waste workers and emergency responders.

Over the past 16 years, the WETP supported its core program, the Hazardous Waste Worker Training Program, by providing over a million workers in all regions of the country with health and safety training. Since 1986, the scope of the program expanded to include the following grant activities:

- the Department of Energy (DOE) Nuclear Training Program—awardees trained nearly 175,000 environmental response and cleanup workers at the DOE Nuclear Weapons Complex;
- the Minority Worker Training Program—awardees successfully trained more than 2,600 young minority adults in worker health and safety for construction and environmental cleanup;
- the Brownfields Minority Worker Training Program—awardees trained nearly 2,000 workers in 15 brownfields communities, in the process positively changing the lives of the trainees and their families in many different ways; and
- the Small Business Innovation Research E-Learning Program—awardees created e-learning technology that supports high-quality health and safety training for hazardous waste workers and emergency responders.

In addition to these activities, since 11 September 2001 the WETP has also trained workers in cleaning up environmental problems stemming from the World Trade Center attacks, as well as potential bioterrorism and use of weapons of mass destruction.

**WETB Staff**

**Joseph Hughes, Jr.—PROGRAM DIRECTOR** | hughes3@niehs.nih.gov

Hazardous Waste Worker Training, DOE Nuclear Training

**Sharon Beard—INDUSTRIAL HYGIENIST** | beard1@niehs.nih.gov

Hazardous Waste Worker Training, DOE Nuclear Training, Minority Worker Training, Brownfields Minority Worker Training

**Ted Outwater—PUBLIC HEALTH EDUCATOR** | outwater@niehs.nih.gov

Small Business Innovation Research E-Learning

**Patricia Thompson—PROGRAM ANALYST** | thompso2@niehs.nih.gov

## Figures and Tables

**Figure:**